# Developing an alcohol policy assessment toolkit: application in the western Pacific

**DOI:** 10.2471/BLT.13.130708

**Published:** 2014-06-23

**Authors:** Natacha Carragher, Joshua Byrnes, Christopher M Doran, Anthony Shakeshaft

**Affiliations:** a National Drug and Alcohol Research Centre, University of New South Wales, 22-32 King Street, Sydney, New South Wales, 2031, Australia.; b Centre for Applied Health Economics, Griffith University, Meadowbrook, Australia.; c Hunter Medical Research Institute, New Lambton, Australia.

## Abstract

**Objective:**

To demonstrate the development and feasibility of a tool to assess the adequacy of national policies aimed at reducing alcohol consumption and related problems.

**Methods:**

We developed a quantitative tool – the Toolkit for Evaluating Alcohol policy Stringency and Enforcement (TEASE-16) – to assess the level of stringency and enforcement of 16 alcohol control policies. TEASE-16 was applied to policy data from nine study areas in the western Pacific: Australia, China excluding Hong Kong Special Administrative Region (SAR), Hong Kong SAR, Japan, Malaysia, New Zealand, the Philippines, Singapore and Viet Nam. Correlation and regression analyses were then used to examine the relationship between alcohol policy scores and income-adjusted levels of alcohol consumption per capita.

**Findings:**

Vast differences exist in how alcohol control policies are implemented in the western Pacific. Out of a possible 100 points, the nine study areas achieved TEASE-16 scores that ranged from 24.1 points for the Philippines to 67.5 points for Australia. Study areas with high policy scores – indicating relatively strong alcohol policy frameworks – had lower alcohol consumption per capita. Sensitivity analyses indicated scores and rankings for each study area remained relatively stable across different weighting schemes, indicating that TEASE-16 was robust.

**Conclusion:**

TEASE-16 could be used by international and national regulatory bodies and policy-makers to guide the design, implementation, evaluation and refinement of effective policies to reduce alcohol consumption and related problems.

## Résumé

**Objectif:**

Démontrer le développement et la faisabilité d'un outil pour évaluer l'adéquation des politiques nationales visant la réduction de la consommation d'alcool et des problèmes associés.

**Méthodes:**

Nous avons développé un outil quantitatif permettant d'évaluer le niveau de sévérité et d'application de 16 politiques de lutte contre l'alcoolisme (TEASE-16 pour «Toolkit for Evaluating Alcohol policy Stringency and Enforcement»). TEASE-16 a été appliqué aux données des politiques issues de 9 zones d'étude dans le Pacifique occidental: l'Australie, la Chine en excluant la Région administrative spéciale (RAS) de Hong Kong, la RAS de Hong Kong, le Japon, la Malaisie, la Nouvelle-Zélande, les Philippines, Singapour et le Viet Nam. Des analyses de corrélation et de régression ont ensuite été utilisées pour examiner la relation entre les résultats des politiques en matière d'alcool et les niveaux de consommation par habitant, ajustés en fonction du revenu.

**Résultats:**

D'énormes différences existent dans la manière de mettre en œuvre les politiques de lutte contre l'alcoolisme dans le Pacifique occidental. Sur un total de 100 points possibles, les 9 zones d'étude ont obtenu des résultats de TEASE-16 compris entre 24,1 points pour les Philippines et 67,5 points pour l'Australie. Les zones d'étude avec des résultats de politique élevés – indiquant des cadres juridiques en matière d'alcool relativement sévères – présentaient une consommation d'alcool par habitant plus faible. Les analyses de sensibilité indiquaient que les résultats et les classements pour chaque zone d'étude restaient relativement stables avec toutes les différentes méthodes de pondération, indiquant que TEASE-16 était fiable.

**Conclusion:**

TEASE-16 pourrait être utilisé par les organismes de réglementation et les responsables politiques internationaux et nationaux pour guider la conception, la mise en œuvre, l'évaluation et l'amélioration des politiques en vigueur pour réduire la consommation d'alcool et les problèmes associés.

## Resumen

**Objetivo:**

Demostrar el desarrollo y la viabilidad de un instrumento para evaluar la adecuación de las políticas nacionales destinadas a reducir el consumo de alcohol y los problemas relacionados con este.

**Métodos:**

Hemos desarrollado una herramienta cuantitativa - Toolkit for Evaluating Alcohol policy Stringency and Enforcement (TEASE-16) - para evaluar el nivel de rigor y de cumplimiento de 16 políticas de control del alcohol. Se aplicó el TEASE-16 a datos de políticas de nueve áreas de estudio en el Pacífico Occidental: Australia, China, excepto la Región Administrativa Especial (RAE) de Hong Kong, RAE Hong Kong, Japón, Malasia, Nueva Zelandia, Filipinas, Singapur y Viet Nam. Se emplearon análisis de correlación y regresión para examinar la relación entre las puntuaciones de las políticas relativas al alcohol y los niveles ajustados por ingresos de consumo de alcohol per cápita.

**Resultados:**

El modo de implementar las políticas de control del alcohol en el Pacífico Occidental presenta grandes diferencias. De los 100 puntos posibles, las nueve áreas de estudio lograron puntuaciones TEASE-16 que variaron de 24,1 puntos para Filipinas a 67,5 puntos para Australia. Las áreas de estudio cuyas políticas obtuvieron los resultados más altos, que indican marcos normativos relativos para el alcohol relativamente sólidos, presentaron un consumo de alcohol per cápita menor. Los resultados y clasificaciones de los análisis de sensibilidad para cada área de estudio se mantuvieron relativamente estables en distintos sistemas de ponderación, lo que indica que TEASE-16 es una herramienta sólida.

**Conclusión:**

Los organismos reguladores internacionales y nacionales y legisladores podrían utilizar TEASE-16 para orientar el diseño, la ejecución, la evaluación y el perfeccionamiento de políticas eficaces para reducir el consumo de alcohol y los problemas relacionados.

## ملخص

**الغرض:**

شرح تطوير إحدى الأدوات وجدواها بغية تقييم ملائمة السياسات الوطنية الرامية للحد من استهلاك الكحول والمشكلات ذات الصلة به.

**الطريقة:**

طورنا أداة كمية - مجموعة أدوات لتقييم الصرامة والإنفاذ في السياسات الخاصة بالكحول (TEASE-16) - لتقييم مستوى صرامة وإنفاذ سياسات مكافحة الكحول الست ة  عشرة. وتم تطبيق TEASE-16 على بيانات السياسة المستمدة من تسع مناطق دراسية في غرب المحيط الهادئ، هي: أستراليا والصين باستثناء إقليم هونغ كونغ الإداري الخاص (SAR) وإقليم هونغ كونغ الإداري الخاص واليابان وماليزيا ونيوزيلندا والفلبين وسنغافورة وفييت نام. وبعد ذلك تم استخدام تحليلات الارتباط والارتداد لدراسة العلاقة بين درجات السياسات الخاصة بالكحول ومستويات استهلاك الكحول للفرد المصححة باحتساب الدخل.

**النتائج:**

توجد اختلافات كبيرة في طريقة تنفيذ سياسات مكافحة الكحول في غرب المحيط الهادئ. ومن أصل 100 نقطة ممكنة، حققت المناطق الدراسية التسع درجات TEASE-16 تراوحت من 24.1 نقطة للفلبين إلى 67.5 نقطة لأستراليا. وانخفض استهلاك الكحول للفرد في مناطق الدراسة التي حصلت على درجات عالية فيما يتعلق بالسياسات - مما يشير إلى أطر سياسات كحول قوية نسبياً. وأشارت تحليلات الحساسية إلى بقاء الدرجات والمراتب في كل منطقة من مناطق الدراسة ثابتة نسبياً عبر مخططات الترجيح المختلفة، مما يشير إلى أن TEASE-16 كانت قوية.

**الاستنتاج:**

تستطيع الهيئات التنظيمية الدولية والوطنية وصانعو السياسات استخدام TEASE-16 لتوجيه تصميم سياسات فعالة وتنفيذها وتقديرها وتحسينها بغية الحد من استهلاك الكحول والمشكلات ذات الصلة به.

## 摘要

**目的:**

证明工具的开发和可行性,以评估旨在减少酒精消费和相关问题的国家政策是否充分。

**方法:**

我们开发了一个定量工具(评估酒精政策严格性和实施的工具包,TEASE-16)来评估16项酒精控制政策的严格性和实施水平。对西太平洋九个研究区域的政策数据使用TEASE-16:澳大利亚、中国(不包括香港特别行政区(SAR))、香港特别行政区、日本、马来西亚、新西兰、菲律宾、新加坡和越南。然后使用相关性和回归分析来检查酒精政策分数和人均酒精消费收入调节水平之间的关系。

**结果:**

在西太平洋地区如何实施酒精控制政策存在巨大差异。在可能的100分中,九个研究区域的得分范围从菲律宾的24.1分到澳大利亚的67.5分不等。研究政策得分高的地区(表示相对严厉的酒精政策框架)人均饮酒量较低。敏感性分析显示每一个研究区域在不同加权方案中的分数和排名保持相对稳定,这表明TEASE-16是可靠的。

**结论:**

国际和国家监管机构和政策制定者可以使用TEASE-16来指导设计、实施、评价和优化有效的政策以减少酒精消费和相关问题。

## Резюме

**Цель:**

Продемонстрировать разработку и реализуемость инструмента для оценки пригодности национальных политик, направленных на сокращение потребления алкоголя и связанных с этим проблем.

**Методы:**

Был разработан количественный инструмент – Набор средств для оценки строгости и реализации алкогольной политики (TEASE-16) – для оценки уровня строгости и реализации 16 различных политик по противодействию потреблению алкоголя. Набор инструментов TEASE-16 был применен к данным по реализации политики в девяти исследуемых регионах западной части Тихого океана: Австралии, Китае, за исключением Специального административного района Гонконг (САР Гонконг), Японии, Малайзии, Новой Зеландии, Филиппин, Сингапура и Вьетнама. Затем, с помощью корреляционного и регрессионного анализов было изучено отношение между оценками политики и уровнями потребления алкоголя на душу населения с поправкой на уровень доходов.

**Результаты:**

Существуют значительные различия в реализации политик по противодействию потреблению алкоголя в западной части Тихого океана. Из возможных 100 баллов набранные девятью регионами оценки TEASE-16 варьировались в диапазоне от 24,1 баллов (Филиппины) до 67,5 баллов (Австралия). Исследуемые регионы, получившие высокие оценки политики — указывающие на относительно жесткие рамки политики по противодействию потреблению алкоголя, — имели более низкое потребление алкоголя на душу населения. Анализы чувствительности показали, что оценки и рейтинги для каждого исследуемого региона оставались относительно стабильными при использовании различных средневзвешенных схем подсчета, что указывает на функциональность TEASE-16.

**Вывод:**

TEASE-16 мог бы использоваться международными и национальными регулирующими и директивными органами для разработки, реализации, оценки и уточнения эффективных политик по снижению потребления алкоголя и связанных с этим проблем.

## Introduction

Globally, alcohol consumption contributes to an estimated 9.6% of all disability-adjusted life-years.^1^ Fortunately, effective evidence-based policies for alcohol control can protect population health and well-being, save lives, reduce health-care costs and increase productivity.^2^^–^^4^ Indeed, every European country has some form of national alcohol control policy framework.^3^

Policy development, however, is only one step. The World Health Organization (WHO) and other leading international agencies have repeatedly called for countries to assess, compare and refine their national alcohol control policy frameworks.^3^^,^^5^^,^^6^ There is limited guidance, however, on how these activities should be carried out. A reliable and valid tool for quantifying alcohol policy stringency and enforcement would yield a standardized, succinct summary of a country’s policy framework and facilitate investigation of the relationship between alcohol policies and consumption per capita. It would also enable meaningful comparisons across countries and jurisdictions and comparisons within countries over time. Further, it could highlight weak policies and provide estimates of the impact of policy improvements on consumption.

A small number of scales have been developed to assess national alcohol control policies.^7^ Although informative, these scales have several limitations. These include the failure to: (i) comprehensively assess enforcement,^7^^–^^14^ (even though enforcement varies considerably across policies and countries and is likely to impact effectiveness);^15^ (ii) demonstrate the scale’s feasibility through a practical application;^10^ (iii) demonstrate the scale’s robustness through sensitivity analyses;^7^^–^^12^ (iv) rank countries according to their degree of alcohol control;^12^ (v) relate alcohol policy scores to corresponding per capita consumption estimates^7^^–^^12^ or adjust for income – which shows considerable between-country variation – in per capita consumption estimates;^13^^,^^14^ and (vi) provide complete assessment of policies and use up-to-date literature.^13^^,^^15^^,^^16^ We aimed to address these limitations and develop a comprehensive and practical tool to measure the stringency and enforcement of national alcohol control policies.

To demonstrate our tool’s feasibility and practical value, we applied it to nine study areas in the western Pacific. Despite recent increases in alcohol consumption in the region,^17^ previous alcohol policy scales have been largely applied to Europe. Only two previous studies have evaluated alcohol control policies in the western Pacific and both focused on high-income countries.^12^^,^^13^ In this paper, we compared the relative strength of national alcohol policy frameworks across a range of developed and developing study areas in the region. We used up-to-date policy data, conducted comprehensive sensitivity analyses to demonstrate the tool’s robustness and investigated the relationship between alcohol policy scores and income-adjusted levels of alcohol consumption per capita.

## Methods

### The assessment tool

We developed the Toolkit for Evaluating Alcohol policy Stringency and Enforcement-16 (TEASE-16), which builds on previous policy evaluation scales.^13^ It is the first tool to assess levels of stringency and enforcement comprehensively. As summarized in Table 1, TEASE-16 has five main components: (i) five regulatory domains; (ii) 16 evidence-based alcohol control policies or policy topics; (iii) effectiveness star ratings (i.e. ratings of the effectiveness of the policies in reducing the adverse effects of alcohol, which were based on expert reviews of the literature);^2^ (iv) level of stringency; and (v) level of enforcement. Further details on the conceptual framework of TEASE-16 are provided in Appendix A (available at: https://ndarc.med.unsw.edu.au/resource/appendix-tease-16-supplementary-details).

**Table 1:** Components of the Toolkit for Evaluating Alcohol policy Stringency and Enforcement-16 (TEASE-16)

Domain, policy topic	Effectiveness star rating^a^	Level of stringency	Level of enforcement^b^
**Physical availability**			
Legal minimum age for alcohol purchase (years)	3	16	Poor, moderate, or strong
		17	
		18	
		19	
		≥ 20	
Alcohol server liability for damages caused by actions of patrons	2	No	Poor, moderate or strong
		Yes	
Government monopoly of alcohol retail sales	2	None	Poor, moderate or strong
		Partial government monopoly	
		Full government monopoly	
Restrictions on density of outlets	2	None	Poor, moderate or strong
		On wine only	
		On wine and spirits	
		On wine, spirits and beer	
Restrictions on the hours and days of sale	2	None	Poor, moderate or strong
		On hours or days	
		On both hours and days	
**Drinking context**			
Community mobilization programmes to increase public awareness or prevent alcohol problems	2	No	Poor, moderate or strong
		Yes	
Mandatory training of bar staff and management to better manage aggression	2	No	Poor, moderate or strong
		Yes	
**Alcohol prices**^c^			
Beer price index	3	0–0.29	Poor, moderate or strong
		0.30–0.59	
		0.60–0.89	
		≥ 0.90	
Wine price index	3	0–0.9	Poor, moderate or strong
		1.0–1.9	
		2.0–2.9	
		≥ 3.0	
Spirit price index	3	0–2.9	Poor, moderate or strong
		3.0–5.9	
		6.0–8.9	
		≥ 9.0	
**Alcohol advertising**			
Restrictions imposed on the majority of alcohol advertising media	1	No restrictions	Poor, moderate or strong
		Industry self-regulation	
		Partial statutory restrictions	
		Ban	
**Drivers of motor vehicles**			
Frequency of random breath testing	3	Never	Poor, moderate or strong
		Rarely	
		Occasionally	
		Often	
		Very often	
Legal blood alcohol concentration limit in adult drivers (mg/dL)	3	≥ 0.08	Poor, moderate or strong
		0.03–0.07	
		0–0.02	
Legal blood alcohol concentration limit in youth drivers (mg/dL)	3	≥ 0.04	Poor, moderate or strong
		0.02–0.03	
		0–0.01	
Mandatory penalties for exceeding the legal maximum blood alcohol concentration	2	No penalty	Poor, moderate or strong
		Fine	
		Penalty points	
		Disqualification or licence suspension	
		Imprisonment	
		Other	
Graduated licensing for young drivers	2	No	Poor, moderate, or strong
		Yes	

^a^ Policies that were considered to be effective in reducing the adverse effects of alcohol were given one-, two- and three-star ratings, respectively.^2^

^b^ Poor reflects policies that were rarely or poorly enforced, or instances where no legislation or no enforceable powers were in place; moderate refers to policies that had limited or occasional enforcement, or were enforced only when violations were reported or blatant; and strong reflects widely enforced policies.

^c^ Price indexes were calculated using guidelines by Brand et al.^13^ The price index refers to the retail price (including alcohol taxes) for a standard size beverage container (0.5 L beer, 0.75 L wine or 0.75 L spirits), taking into account a country’s standard of living. This adjustment involves dividing the retail price by the per capita share of a country’s gross domestic product and subsequently multiplying the result by 10 000 to yield a price index with an approximate range of 0–20.

Following Brand et al.,^13^ we examined five broad regulatory domains that were identified in a WHO-sponsored comprehensive analysis of alcohol policies.^2^ Within these domains, we focused on 16 policies that have been implemented around the world and evaluated by experts as being effective in reducing the adverse effects of alcohol.^2^ Like Brand et al.,^13^ we excluded policies that: have limited effectiveness (e.g. warning labels on containers for alcoholic drinks and/or that relate to the treatment of problem drinkers) because we wished to focus on preventive public health strategies; and we were not implemented in any of the nine study areas under investigation (e.g. minimum pricing).

Each policy was rated according to level of stringency and enforcement (Appendix A). Briefly, stringency refers to the relative strictness of a given policy. For example, limiting the age of those who can purchase alcohol to 16, 17, 18, 19 or ≥ 20 year-olds, reflects increasingly stringent policy positions on controlling the availability of alcohol. Enforcement refers to the strength at which a given policy is implemented in practice. We divided levels of enforcement into three categories: (i) poor – reflecting policies that were rarely or poorly enforced, or instances where no legislation or enforceable powers were in place; (ii) moderate – referring to policies that had limited or occasional enforcement or were enforced only when violations were reported or blatant; and (iii) strong – reflecting widely enforced policies.

Although TEASE-16 builds on Brand et al.’s Alcohol Policy Index,^13^ the scales differ in terms of policy conceptualizations, effectiveness ratings, inclusion of enforcement ratings, the development of three alternative weighting schemes combining stringency and enforcement ratings, and the use of income-adjusted estimates of alcohol consumption per capita (Appendix A).

### Scoring and sensitivity analyses

Each of the 16 policy topics was allocated a maximum potential number of points based on peer-reviewed assessments of effectiveness in reducing the adverse effects of alcohol (Table 1). Proportionate points were then allocated according to the particular level of stringency and enforcement. For each study area, scores across all 16 policy topics were collated to yield an overall score ranging between 0 and 100. Then study areas were rank ordered.

To examine the robustness of TEASE-16, we applied different weighting methods to each policy topic according to its effectiveness rating and subsequently calculated the corresponding proportionate point scores. We tested alternative weighting methods to avoid the risk of study areas rejecting TEASE-16 on the grounds that a particular weighting scheme was unfairly punitive.

In total, four different weighting methods were used to assign stringency and enforcement points: baseline, heavy, equal and area-specific. In baseline weighting, weights of 1, 2 and 3 were applied to policy topics with one-, two- and three-star effectiveness ratings, respectively. Heavy weighting used corresponding weights of 1, 3 and 5 whereas equal weighting assigned the same weight to all policies regardless of effectiveness ratings. Area-specific weights were also derived – using data envelopment analysis^18^ and implemented with the Solver add-in for Excel 2010 (Microsoft, Redmond, United States of America) – in a manner that optimized a study area’s relative performance. Area-specific weights were constrained to reflect effectiveness ratings (i.e. a three-star policy topic received a greater weight than a two-star policy and a two-star topic received a greater weight than a one-star policy). Additional constraints were specified to ensure that the area-specific weights were plausible and to avoid instances where a study area might be awarded a perfect rating because zero weights had been allocated to policy topics that had minimal stringency. For example, the area-specific weights were constrained so that the maximum weight was less than 12-fold higher than the minimum weight.

For each of the four weighting schemes, three methods were used to yield combined ratings for stringency and enforcement (see Appendix A). In one method – 50:50 combination – equivalent points were allocated to stringency and enforcement. In another method – 25:75 combination – a quarter of the points were assigned to stringency and the remainder to enforcement. In the third method – multiplicative combination – stringency ratings were multiplied by a third of the raw enforcement rating. All calculations were conducted in Excel 2010.

### Data retrieval

Since low-income nations generally have a greater disease burden per unit of alcohol consumption than high-income nations,^19^ we retrieved alcohol policy and consumption data from both developed areas of the western Pacific (Australia, China excluding Hong Kong Special Administrative Region (SAR), Hong Kong SAR, Japan, New Zealand and Singapore) and developing areas (Malaysia, the Philippines and Viet Nam).^20^ These nine study areas are economically diverse and geographically widespread; they have different epidemiological profiles and reflect a range of cultural, religious and social practices relating to alcohol use.

#### Policies

We obtained data on stringency and enforcement from peer-reviewed papers and WHO reports published between 2008 and 2012, as well as government and related public health websites. A full list of the data sources is available from the corresponding author. Where information was unclear or outdated, we verified policies with the relevant public health and government officials in August–October 2012. This ensured that we included the most up-to-date legislation and that policy topics were correctly weighted. Extensive efforts were undertaken to cross-reference data to ensure accuracy. Complete information on stringency and enforcement was retrieved for all 16 policy topics in each of the nine study areas.

#### Alcohol consumption

For each study area, an estimate of the average percentage of alcohol by volume was used to convert total volume of alcoholic drinks sold in 2011^21^^–^^29^ into total volume of alcohol consumed in pure alcohol. The result was then multiplied by 1 000 000 and divided by the population estimate, in millions, for the study area– obtained via the websites of the relevant national statistics agency and verified by officials – to yield an estimate of the mean volume of alcohol consumed per capita in 2011. Since alcohol consumption is positively related to income,^2^ we divided each estimate by the relevant gross domestic product per capita – reported in international dollars using purchasing power parity exchange rates^30^ – to yield an income-adjusted estimate of alcohol consumption per capita for the year 2011 in each study area.

### Analysis

For each study area, we calculated alcohol policy scores using 12 sets of assumptions – reflecting the four weighting methods and three methods for combining ratings of stringency and enforcement. To facilitate comparisons, we calibrated the scores generated under each set of assumptions to yield equivalent ranges. Subsequently, we identified the median rank and overall score for all 12 assumptions for each study area. We then calculated Pearson’s or Spearman’s correlation coefficients – as appropriate – to compare these medians with the corresponding baseline ranks (i.e. those produced using baseline weighting). Additionally, we calculated correlations using the extreme values – rather than medians – to provide a measure of the robustness of TEASE-16. To evaluate the relationship between policy scores and income-adjusted alcohol consumption per capita, we performed a simple linear regression in SPSS (SPSS Inc., Chicago, USA).

## Results

### Strength of policy frameworks

We compared the comprehensiveness of alcohol control policies in nine study areas in the western Pacific by calculating a rating for each regulatory domain (Table 2). Overall, the median rating was 56.4 points – out of a possible 100 points – with ratings ranging from 24.1/100 in the Philippines to 67.5/100 in Australia. The nine study areas received median domain ratings of 5.9/28.9 points for physical availability policies, 3.9/10.5 points for drinking context policies, 18.4/23.7 points for alcohol pricing policies, 0.4/2.6 points for alcohol advertising policies and 23/34.2 points for motor vehicle regulations.

**Table 2:** TEASE-16 alcohol policy scores for nine study areas in the western Pacific, 2011

Study area	Rank	Points scored					
		Physical availability	Drinking context	Alcohol prices	Alcohol advertising	Motor vehicle regulations	Total
Australia	1	11.2	5.3	18.4	0.4	32.2	67.5
Singapore	2	14.5	5.3	23.7	0.4	20.5	64.4
New Zealand	3	3.9	3.9	23.7	0.4	30.3	62.3
Hong Kong SAR	4	10.5	5.3	17.8	1.5	23.0	58.1
Japan	5	5.9	3.9	21.1	0.4	25.0	56.4
Malaysia	6	9.6	3.9	23.7	2.0	16.6	55.8
China^a^	7	5.9	0.0	17.8	0.0	26.4	50.1
Viet Nam	8	5.9	7.9	11.8	2.6	13.6	41.8
Philippines	9	5.9	0.0	17.8	0.4	0.0	24.1
**Median**		**5.9**	**3.9**	**18.4**	**0.4**	**23.0**	**56.4**
**Maximum points available**		**28.9**	**10.5**	**23.7**	**2.6**	**34.2**	**100**

SAR: Special Administrative Region; TEASE-16: Toolkit for Evaluating Alcohol policy Stringency and Enforcement-16.

^a^ Excluding Hong Kong SAR.

Note: Low scores indicate scope for strengthening policies.

### Policy scores and alcohol consumption

**Fig. 1:**
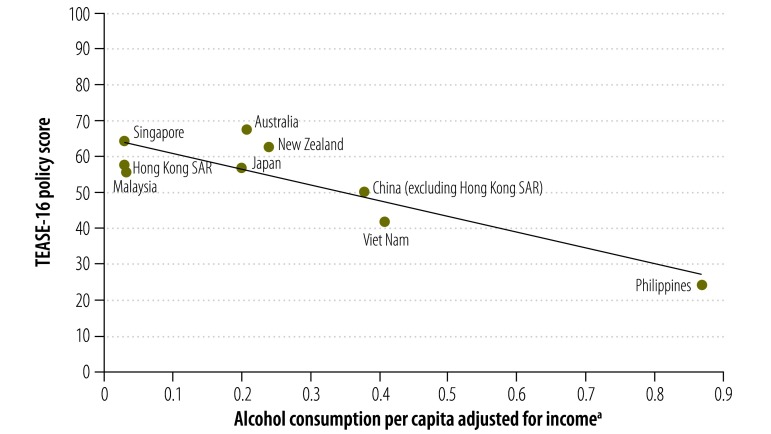
Relationship between alcohol policy scores and income-adjusted alcohol consumption per capita, western Pacific, 2011

GDP: gross domestic product; SAR: Special Administrative Region; TEASE-16: Toolkit for Evaluating Alcohol policy Stringency and Enforcement.

^a^ Litres per 1000 international dollars of GDP per capita.

Note: Policy scores were determined for nine study areas using the TEASE-16.

A strong inverse relationship was observed between income-adjusted levels of alcohol consumption per capita and alcohol policy scores (*r* = −0.88; *P* = 0.001; Fig. 1). To exclude price demand influences, we recalculated alcohol policy scores after removing alcohol prices from the model. This resulted in a minor change to the observed relationship (*r* = −0.83; *P* = 0.003). Based on the slope of the regression line, a one-point increase in alcohol policy score equated to a 1.8% decrease in income-adjusted consumption of alcohol consumption per capita.

### Sensitivity analyses

Rankings remained relatively stable across the 12 assumptions tested, with those of only three study areas – Hong Kong SAR, Japan and Malaysia – differing by three positions (Table 3). Correlation analyses confirmed that median ranks (*r* = 0.98; *P* < 0.0001) and ratings (*r* = 1.0; *P* < 0.0001) differed minimally from the baseline ranks and ratings. Indeed, even when baseline ranks and ratings were compared with the extreme corresponding ranks (*r* = 0.97; *P* < 0.0001) and ratings (*r* = 0.97; *P* < 0.0001) from the 12 assumptions, correlation coefficients remained high. The negative relationship between median ratings and consumption remained robust (*r* = −0.86, *P* = 0.003).

**Table 3:** TEASE-16 sensitivity analyses for nine study areas in the western Pacific, 2011

Study area	Rank^a^				Score^a^		
	Baseline	Median	Range		Baseline	Median	Range
Australia	1	1	1–2		67.5	62.2	36–83
Singapore	2	3	2–4		64.4	56.7	30–80
New Zealand	3	3	1–3		62.3	56.3	32–74
Hong Kong SAR	4	5	4–7		58.1	51.8	24–73
Japan	5	4	3–6		56.4	50.7	26–65
Malaysia	6	6	4–7		55.8	49.9	25–71
China^b^	7	7	5–7		50.1	46.7	22–58
Viet Nam	8	8	8–9		41.8	39.1	11–56
Philippines	9	9	8–9		24.1	20.9	8–26

SAR: Special Administrative Region; TEASE-16: Toolkit for Evaluating Alcohol policy Stringency and Enforcement-16.

^a^ In total, 12 alternative weighting methods were tested. The baseline model generated ranks and scores, whereas the remaining models generated medians and ranges.

^b^ Excluding Hong Kong SAR.

## Discussion

Studies comparing alcohol policy frameworks and consumption across countries are scarce.^15^ To address this gap, we developed and applied a tool to evaluate policies across nine study areas in the western Pacific. We found striking variations in how policies were implemented. Among the nine study areas evaluated, the Philippines had the weakest alcohol policy framework whereas Australia had the strongest. Australia was particularly strong in relation to policies limiting driving while under the influence of alcohol and alcohol pricing policies. In Australia – as in most of the other study areas – alcohol advertising policies were relatively weak. In the Philippines, all regulatory domains were generally weak, particularly those relating to drinking context and driving policies.

Although we used TEASE-16 to evaluate alcohol control policies and consumption at a particular point in time, the tool could be used to evaluate policy changes and consumption within a study area over time. Under a log regression function, an increase in alcohol policy score would have a greater impact on consumption for study areas with weak policy frameworks than for study areas with strong policy frameworks. For example, targeted policy improvements resulting in a seven-point increase in the TEASE-16 score would reduce per capita consumption in the Philippines – per 1000 international dollars of gross domestic product – by 0.19 litres but the corresponding reduction in Japan would only be 0.09 litres.

Like Brand et al.,^13^ we found that study areas with more stringent – and strongly enforced – alcohol policies had significantly lower levels of consumption. Although we recognize that there are many structural and contextual factors influencing the extent and patterns of alcohol consumption, the results indicate that alcohol consumption rates – whatever their causes and even after controlling for differences in income – are at least partially related to the strength of national alcohol control policies.

In addition to the need for ongoing social and treatment programmes for individuals and communities at high risk of alcohol-related harm, WHO encourages its Member States to regularly assess and refine their alcohol control policy frameworks.^4^^,^^6^ This paper highlights considerable scope for strengthening policies in the western Pacific, particularly in relation to the advertising and physical availability of alcohol.

Our study has several limitations. First, considerable heterogeneity exists in alcohol policies and cultural differences may affect the level of alcohol consumption per capita. These differences are not captured in TEASE-16, which focuses on formal, national alcohol policies. Nevertheless, where possible, we attempted to minimize the effects of differences within study areas. For Australia, for example, we collected data from all eight states and territories for each policy topic and then used the general consensus to reflect the national position. Second, although TEASE-16 assesses a panoply of alcohol control policies, it does not cover the full spectrum of policies. However, as Karlsson and Österberg point out,^11^ it would be laborious – if not impossible – to do this, as there are over 100 relevant policies.

Third, it is well recognized in the literature that there is a close relationship between national affluence and alcohol consumption.^31^ For this reason, we adjusted consumption estimates to take account of each study area’s per capita gross domestic product. However, the extent and patterns of alcohol consumption in any nation are not entirely determined by regulatory framework and affluence.^31^^,^^32^ Many other factors (e.g. socioeconomic factors, physical environment, biological and genetic factors, access to health-care services and facilities, and individual characteristics) are involved. Any observed disparities in consumption patterns may therefore result from the complex interplay of a variety of structural and contextual factors. In designing and implementing effective alcohol control policies, it is important to account for this panoply of mitigating factors and to adopt a coordinated response.

Fourth, the cross-sectional nature of this study means that a causal relationship between alcohol policy scores and income-adjusted estimates of per capita alcohol consumption cannot be inferred. Fifth, cross-national comparisons will necessarily restrict sample size because the collection of policy data and the cross-referencing of sources are so time-consuming. The use of small sample sizes reduces statistical power and increases the likelihood of potential bias from outliers. Accordingly, caution should be exercised in extrapolating this study’s findings beyond the study areas examined. However, since our finding of an inverse relationship between alcohol policy scores and alcohol consumption remained robust across 12 alternative weighting schemes – and matches the conclusions drawn by Brand et al.,^13^ who analysed alcohol policies in 30 countries – we can conclude there was little bias in our study.

Sixth, although enforcement is a critical component of policy evaluation, its measurement presents a challenge due to the difficulty in securing objective data. To minimize bias, we attempted to verify enforcement data by cross-referencing information with numerous officials and against relevant statistics. Finally, while TEASE-16 appears to be reliable and have content, face and criterion validity; construct validity and test–retest reliability have yet to be established.

Despite its shortcomings, TEASE-16 has numerous benefits. First, TEASE-16 provides an updated, empirical synopsis of national policies across several study areas. Second, by reducing a vast amount of data into a single score, the tool is useful for facilitating communication with the general public, public health advocates and policy-makers. Third, TEASE-16 overcomes limitations of previous alcohol policy scales. In the future, TEASE-16 could be used to conduct a more nuanced examination of the relationship between targeted policies (e.g. measures taken against driving while under the influence of alcohol) and specific outcomes (e.g. numbers of alcohol-related road traffic accidents and fatalities).

In summary, this paper presents an empirical tool for the comprehensive assessment of the stringency and enforcement of alcohol control policies. TEASE-16 could be employed by national policy-makers and regulatory bodies to identify opportunities for developing or refining national policy frameworks and measuring the impact of policy changes on consumption. If risky alcohol consumption and related harms are to be reduced in the western Pacific, efforts could be targeted towards strengthening weak policies, such as those relating to alcohol advertising.
